# A novel prognostic N^7^-methylguanosine-related long non-coding RNA signature in clear cell renal cell carcinoma

**DOI:** 10.1038/s41598-023-45287-w

**Published:** 2023-10-27

**Authors:** Wang Luo, Jing Lu, Xiang Zheng, JinJing Wang, ShengYan Qian, ZhiXun Bai, MingSong Wu

**Affiliations:** 1 School of Stomatology, Zunyi Medical University, Zunyi, 563000 Guizhou China; 2 Department of Clinical, Zunyi Medical and Pharmaceutical College, Zunyi, 563000 Guizhou China; 3 Department of Medical Genetics, Zunyi Medical University, Zunyi, 563000 Guizhou China; 4 Department of Pathology, Affiliated Hospital of Zunyi Medical University, Zunyi, 563000 Guizhou China; 5 Department of Nephrology, the Second Affiliated Hospital of Zunyi Medical University, Zunyi, 563000 Guizhou China

**Keywords:** Cancer, Computational biology and bioinformatics, Genetics, Immunology

## Abstract

Clear cell renal cell carcinoma (ccRCC) is regulated by methylation modifications and long noncoding RNAs (lncRNAs). However, knowledge of N^7^-methylguanosine (m^7^G)-related lncRNAs that predict ccRCC prognosis remains insufficient. A prognostic multi-lncRNA signature was created using LASSO regression to examine the differential expression of m7G-related lncRNAs in ccRCC. Furthermore, we performed Kaplan–Meier analysis and area under the curve (AUC) analysis for diagnosis. In all, a model based on five lncRNAs was developed. Principal component analysis (PCA) indicated that the risk model precisely separated the patients into different groups. The IC_50_ value for drug sensitivity divided patients into two risk groups. High-risk group of patients was more susceptible to A.443654, A.770041, ABT.888, AMG.706, and AZ628. Moreover, a lower tumor mutation burden combined with low-risk scores was associated with a better prognosis of ccRCC. Quantitative real-time polymerase chain reaction (qRT-PCR) exhibited that the expression levels of LINC01507, AC093278.2 were very high in all five ccRCC cell lines, AC084876.1 was upregulated in all ccRCC cell lines except 786-O, and the levels of AL118508.1 and DUXAP8 were upregulated in the Caki-1 cell line. This risk model may be promising for the clinical prediction of prognosis and immunotherapeutic responses in patients with ccRCC.

## Introduction

Kidney cancer is among the most aggressive type of the urinary system worldwide^1^, ccRCC subtype is the most common pathological classification of renal tumors. Current treatments for ccRCC include partial nephrectomy, radical nephrectomy, targeted drug therapy and immunotherapy. Radiogenomics^2^ and the combination of Radiomics and artificial intelligence (AI)^3^ of ccRCC have contributed in trying to improve diagnosis and treatment. However, 30% of patients experience recurrence or metastasis after surgery^4^. Moreover, ccRCC is resistant to existing therapies such as chemotherapy, interferon immunotherapy (IFN-α), tyrosine kinase inhibitor targeted therapy, and mammalian targets of the rapamycin pathway^5–7^. To better understand the molecular mechanisms of ccRCC and improve early diagnosis and treatment, it remains imperative to identify new diagnostic, therapeutic, and prognostic markers. Interestingly, m^7^G tRNA modification enhances the mRNA translation of oncogenes^8^ and is strongly associated with the development of ccRCC^9^ and multiple tumors.

7-methylguanosine (m^7^G) is a type of RNA methylation and is generated by the methylation of a guanosine base at the N-7 position^10^. Most tRNAs are modified at nucleotide 46^11^ in the variable loop to improve stability. m^7^G modification exerts important effects on liver cancer^12^, cardiovascular disease^13^, clear cell kidney cancer^9^, growth, invasion, and metastasis. Modification of m^7^G and atypical expression of lncRNAs may result in different diseases, including many types of cancer.

Long noncoding RNAs (lncRNAs) are non-protein-coding transcripts longer than 200 nucleotides^14^. Recently, lncRNAs were involved in the progression of malignancies according to other aticle, including kidney cancer^15^, papillary thyroid tumors^16^. However, the relationship between m^7^G-related lncRNAs and ccRCC prognosis was unclear. In this study, we used The Cancer Genome Atlas (TCGA) database, m^7^G, and lncRNA analysis at the molecular level to build a prognostic model for the treatment and prognosis of ccRCC patients.

## Materials and methods

### Data collection

Clear cell renal cell carcinoma patient data (72 normal patients and 539 patients) were obtained from TCGA database (https://portal.gdc.cancer.gov/).

### Identification of m^7^G-related lncRNA

Process of this article, we used TCGA-KIRC database and the R program packages “clusterprofiler” “ggplot2” and “enrichplot”. Gene Ontology (GO) and Kyoto Encyclopedia of Genes and Genomes (KEGG) data were used to analyze the biological roles of differentially expressed m^7^G-associated lncRNAs.

### Construction of the m^7^G-related lncRNA prognostic model

Using cox regression analyses to identify crucial increments with m^7^G-related lncRNAs. Risk calculation formula: y = -0.40*LINC01507 + 1.45*AL118508.1 + 0.33*AC093278.2 + 0.72*DUXAP8 + 1.15*AC084876.1. A hybrid nomogram was constructed using prognostic markers and independent factors for m^7^G-associated lncRNAs from the TCGA-KIRC database. Patients were divided into high- and low-risk groups due to the expression levels of m^7^G-related lncRNAs. The hybrid nomogram map was generated by performing receiver operating characteristic (ROC) analysis to estimate the accuracy of 1-, 3- and 5-year OS of patients.

### Kaplan–Meier (K–M) survival analysis and principal component analysis (PCA)

The accuracy of the risk model was examined by K–M survival curves and PCA methods. Using IC_50_ to recognize patient sensitivity to certain drug treatments. In addition, the TIDE model can assess the performance of different risk groups under immunotherapy.

### Cell culture

Human renal cortex proximal tubule epithelial cells (HK-2) and ccRCC cell lines (769-P, Caki-1, SN12C, UO31, and 786-O) were purchased from the Chinese Academy of Sciences and cultured in RPMI 1640 medium (Gibco, United States) with 10% w/v fetal bovine serum (FBS) (Ausgenes, Australia) at 37 °C and 95% humidity in a 5% CO_2_ cell incubator.

### RNA isolation and quantitative real-time polymerase chain reaction (qRT-PCR)

Total RNA was isolated using RNA Iso Plus reagent (Takara Bio, 9108, China). This material was used for cDNA synthesis using the Prime Script RT reagent kit (Takara Bio, RR037A) according to the manufacturer’s instructions. Gene expression was quantified using TB Green Premix Ex TaqII (Takara Bio, RR820A). Sangon Biotech Co. Ltd. (Shanghai, China) synthesized all the primers for qRT-PCR. Following are the 5'–3' primer sequences for qRT-PCR (Table 1).

**Table 1:** The 5′ to 3′ primer sequences used for qRT-PCR.

Gene ID	Primer F	Primer R
AC093278.2	5′ TGCTTGAACCATGATGCCAGTGAG 3′	5′ CTCAGTGCCAGTGCCTAAGTAAGTC 3′
AL118508.1	5′ TGGATCTCCCTCTGCCCTTTGC 3′	5′ TCTCGGTGCCTGCTGCTTTATTTC 3′
DUXAP8	5′ TGTGGATGGGCAGGATGGAGTC 3’	5’ GAGGCAGGAGAATGGCGTGAAC 3’
LINC01507	5’ AGCCCCTGTTGATTGACTTGTCTTG 3′	5′ TCTCCTTCTCTTGCTGTCTCCTACC 3′
AC084876.1	5′ ATGCTCCTTCCATTCCTCACATGC 3’	5′ GAGTAACACGATTCCCTGCTGACC 3’
H-GAPDH	5’ AGAAGGCTGGGGCTCATTTG 3′	5’ AGGGGCCATCCACAGTCTTC 3′

qRT-PCR was performed on a CFX96 TouchTM fluorescent quantitative PCR instrument (Bio-Rad). The PCR procedure was as follows: 40 cycles of 98 °C for 30 s, 98 °C for 5 s, and 60 °C for 5 s. GAPDH served as the internal reference gene for normalization. 2^−ΔΔCt^ method^17^ was used to calculate the expression levels.

### Statistical analysis

All analyses were conducted using R statistical package (version 4.0.2), and statistical significance was defined as p < 0.05. The chi-square test was used to analyze differences in the proportions of the clinical characteristics. The PCR data was analyzed by GraphPad Prism 8.0 using an independent samples *t*-test.

## Results

### Identification of prognostic m^7^G-related lncRNAs signatures in ccRCC

**Figure 1:**
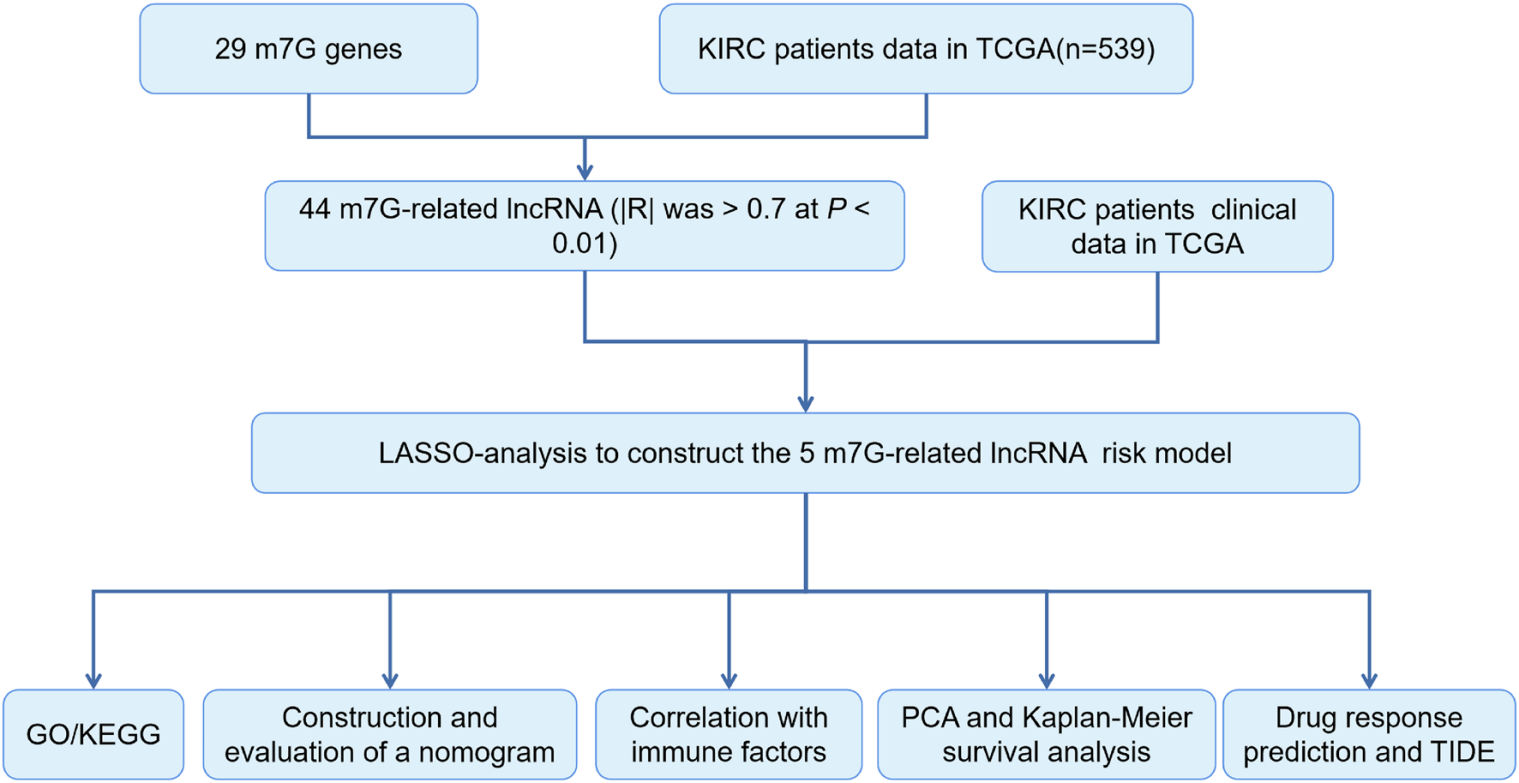
The research process flowchart.

**Figure 2:**
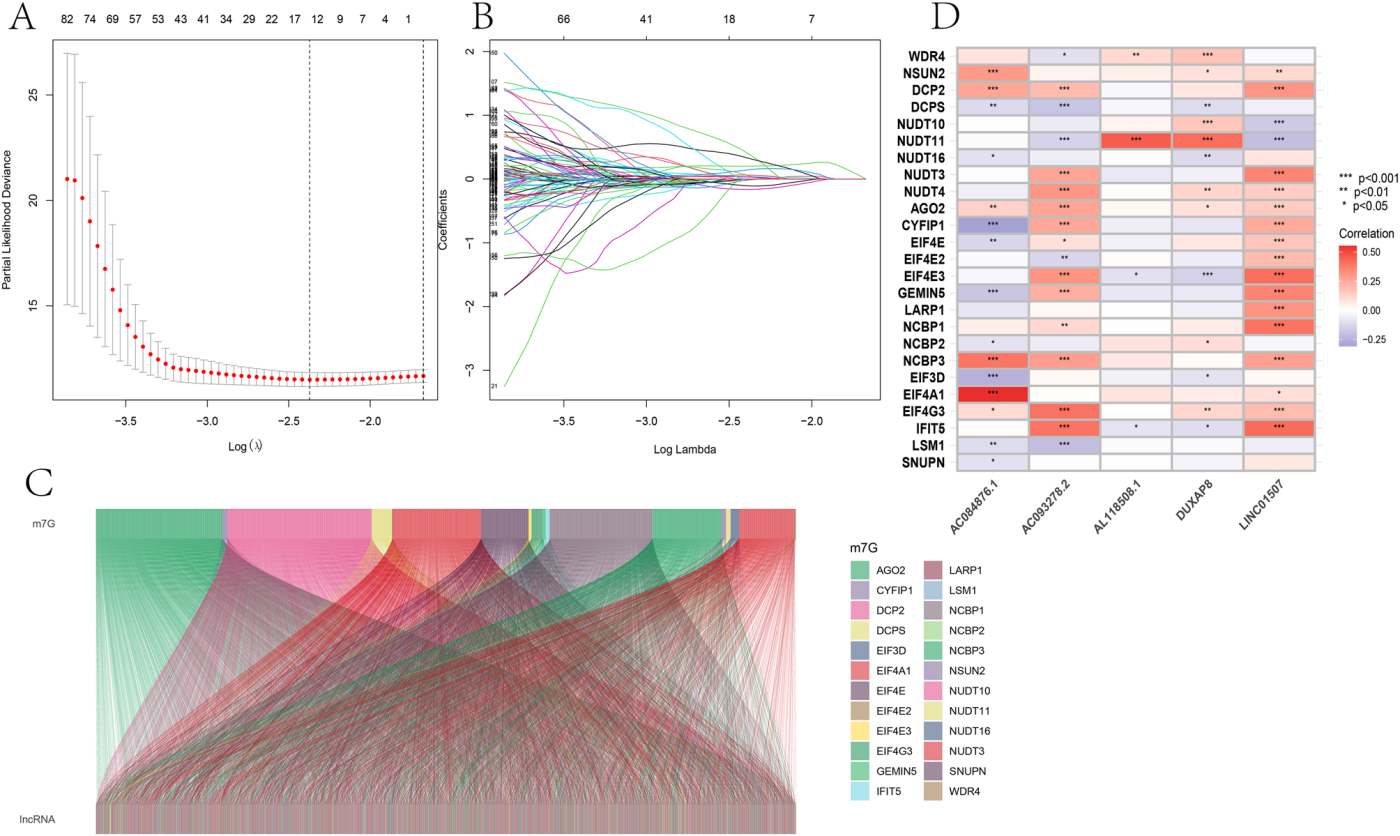
Identification of prognostic m^7^G-related lncRNAs in ccRCC. (**A**) The tuning parameters of LASSO; (**B**) LASSO coefficient profile of m^7^G-related lncRNAs; (**C**) Sankey diagram for m^7^G genes and lncRNAs; (**D**) the correlation heat map for m^7^G genes with 5 m^7^G-related lncRNAs (R program packages, version 4.0.2).

**Figure 3:**
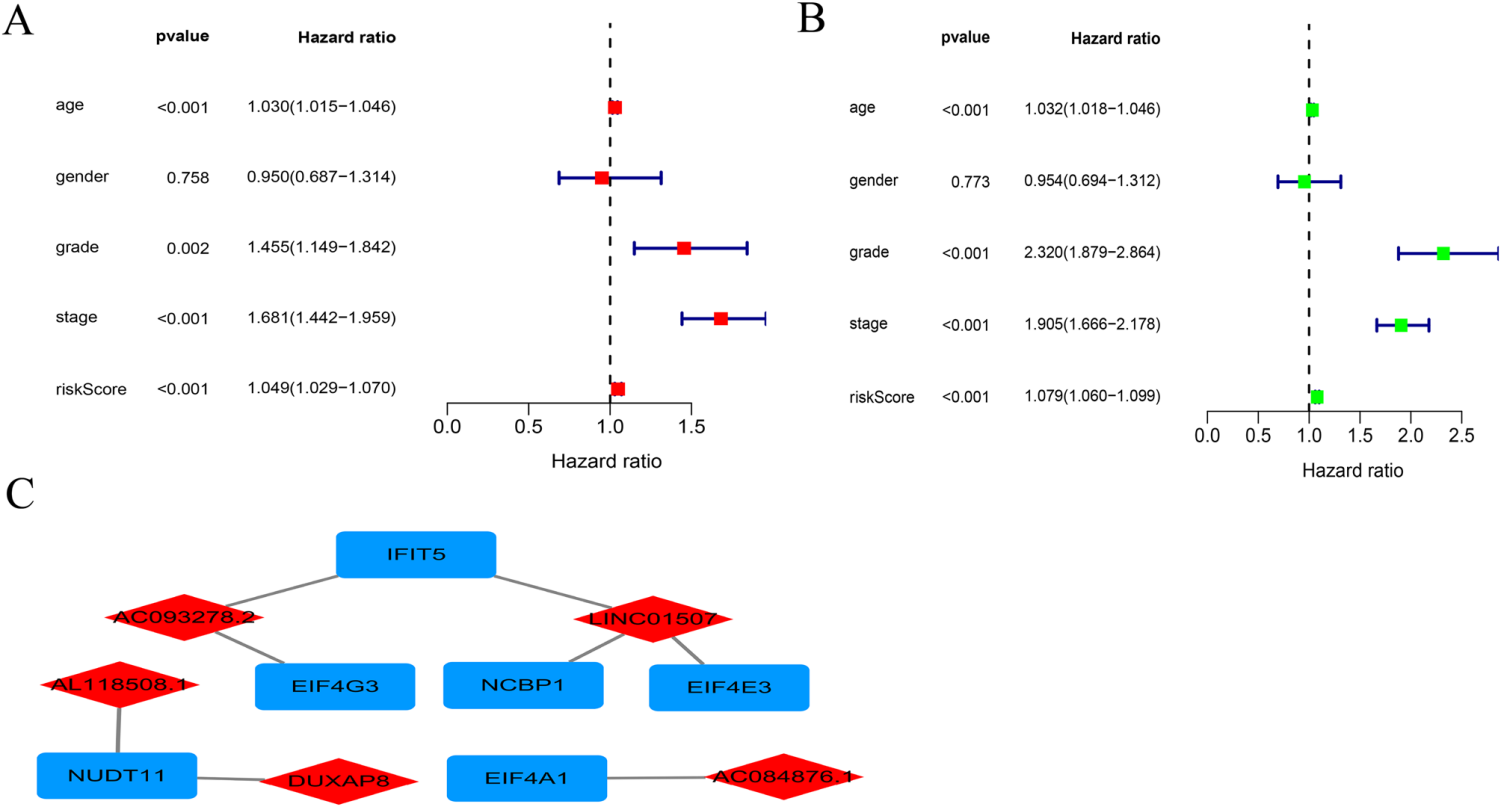
Risk factors of m^7^G-related lncRNAs. (**A**) Multivariate Cox of m^7^G-related lncRNAs; (**B**) univariate Cox of m^7^G-related lncRNAs; (**C**) the correlations between m^7^G-related lncRNAs and mRNA.

In all, 44 m^7^G-related lncRNAs were obtained from 29 m^7^G-related genes combined with the TCGA-KIRC database. Then, comparison of 44 m^7^G-related lncRNAs with KIRC patients clinical information on KIRC provided five m^7^G-related lncRNAs. Based on five m^7^G-related lncRNAs to construct risk model. (Fig. 1). Five differentially expressed lncRNAs (AC084876.1, AC093278.2, AL118508.1, DUXAP8, and LINC01507) were identified by LASSO regression analysis (Fig. 2A, B). Additionally, Sankey plot was used to decipher the relationship between m^7^G-associated differentially expressed genes (DEGs) and lncRNAs (Fig. 2C). Heat map showed the relationship between m^7^G-related genes and m^7^G-related lncRNAs (Fig. 2D). Cox regression analysis indicated that the five m^7^G-associated lncRNAs, grade, age and stage were independent prognostic indicators for the OS of the ccRCC (Fig. 3A, B). Moreover, network of five m^7^G-related lncRNAs is showed in diagram (Fig. 3C).

### Construction of hybrid nomogram and the m^7^G-related lncRNAs set analyses

**Figure 4:**
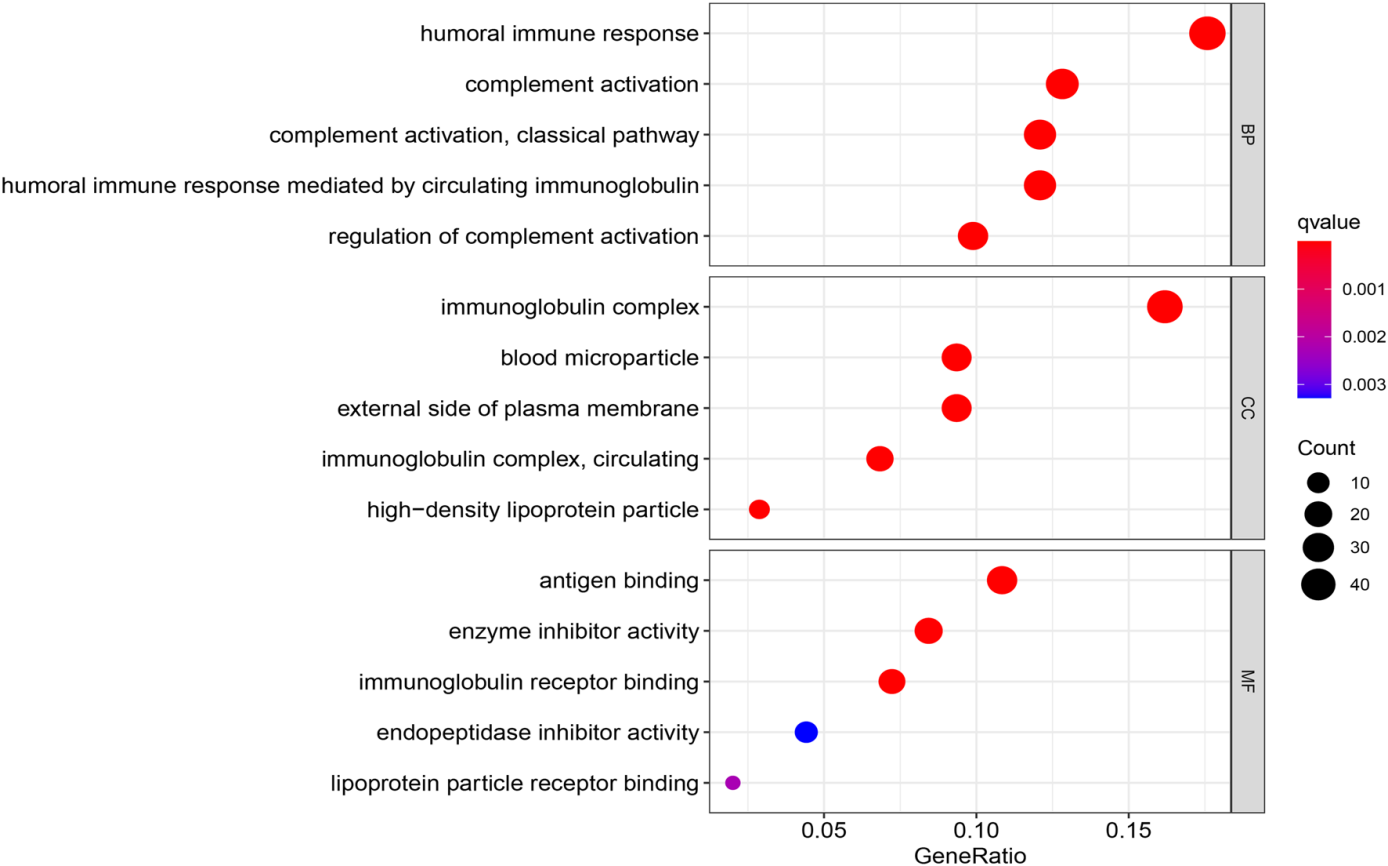
GO enrichment analysis for differentially expressed m^7^G-related genes.

**Figure 5:**
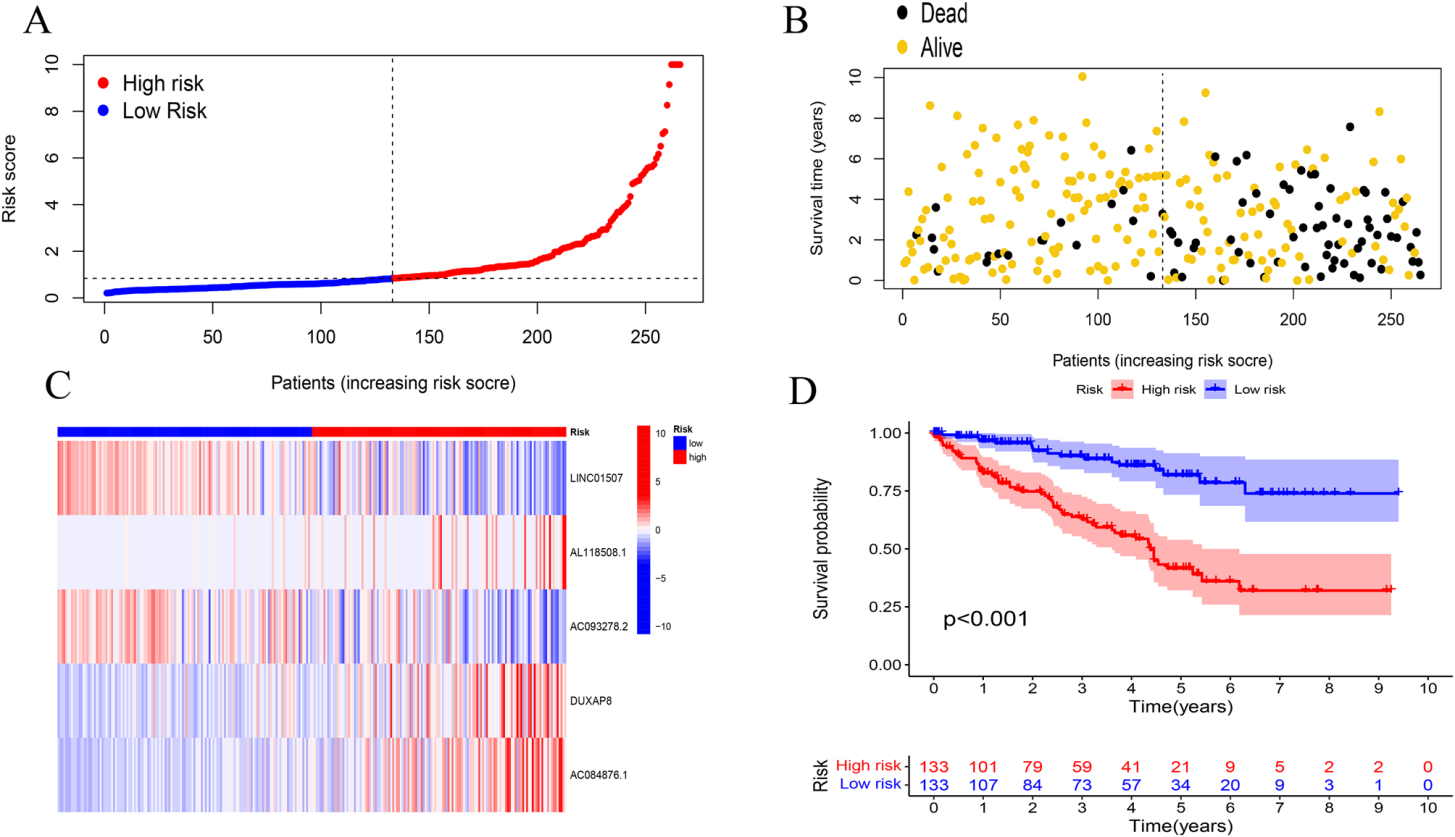
Construction of prognostic model of m^7^G-related lncRNAs; (**A**) the distinct distribution model between two groups of ccRCC; (**B**) the survival status and time in ccRCC patients; (**C**) heat map of the expression level of five m^7^G-related lncRNAs in each patient (R program packages, version 4.0.2); (**D**) overall survival analysis among the high risk and low risk groups.

**Figure 6:**
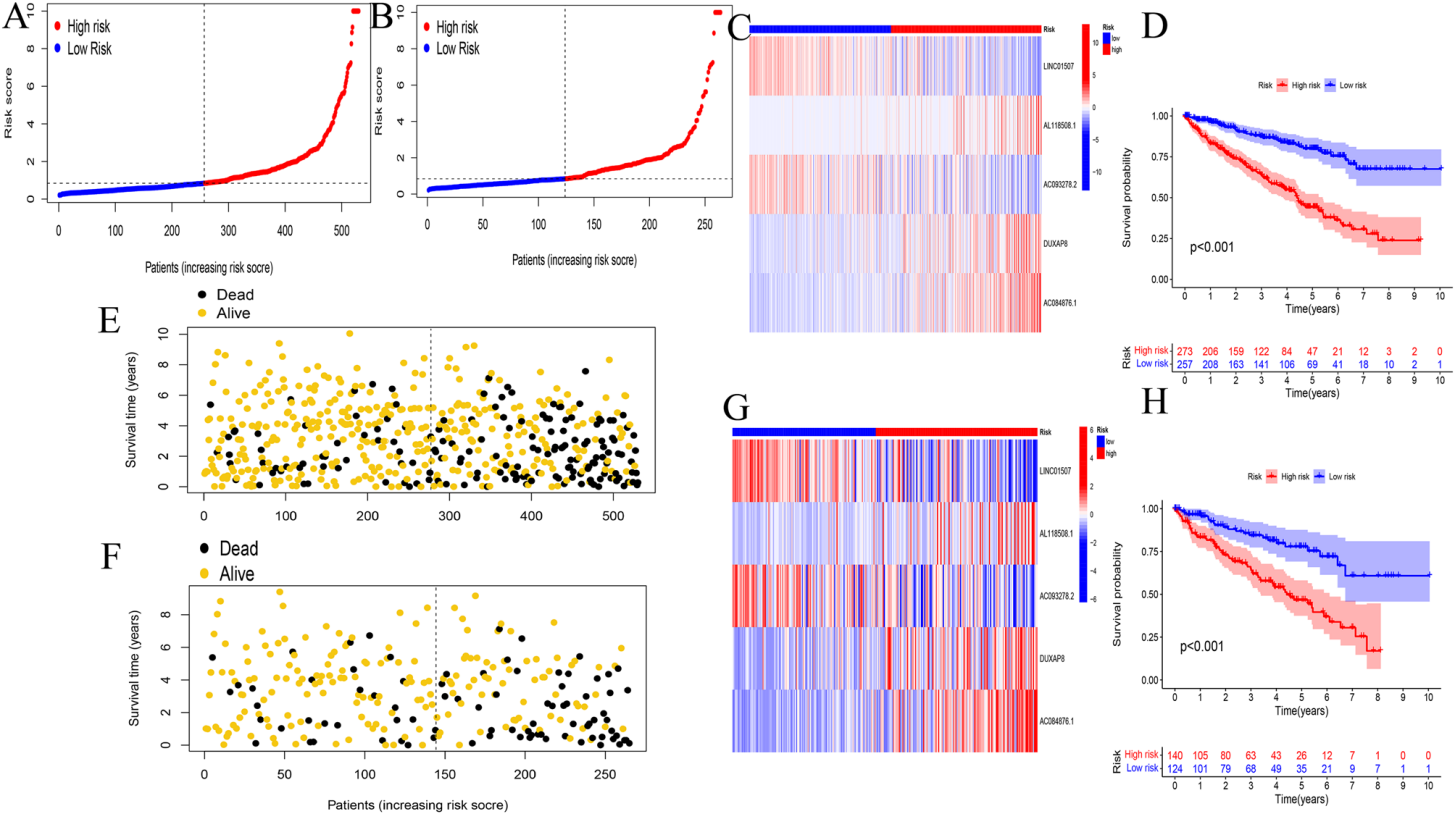
Validation of prognostic model of m^7^G-related lncRNAs. (**A,B**) Distribution of risk scores in the overall group and validation group; (**E,F**) distribution of overall survival status in the overall group and validation group; (**C,G**) heat map of the expression level of the five m^7^G-related lncRNAs in the overall group and validation group (R program packages, version 4.0.2); (**D,H**) Kaplan–Meier curves of overall survival rates of ccRCC patients in the overall group and validation group.

**Figure 7:**
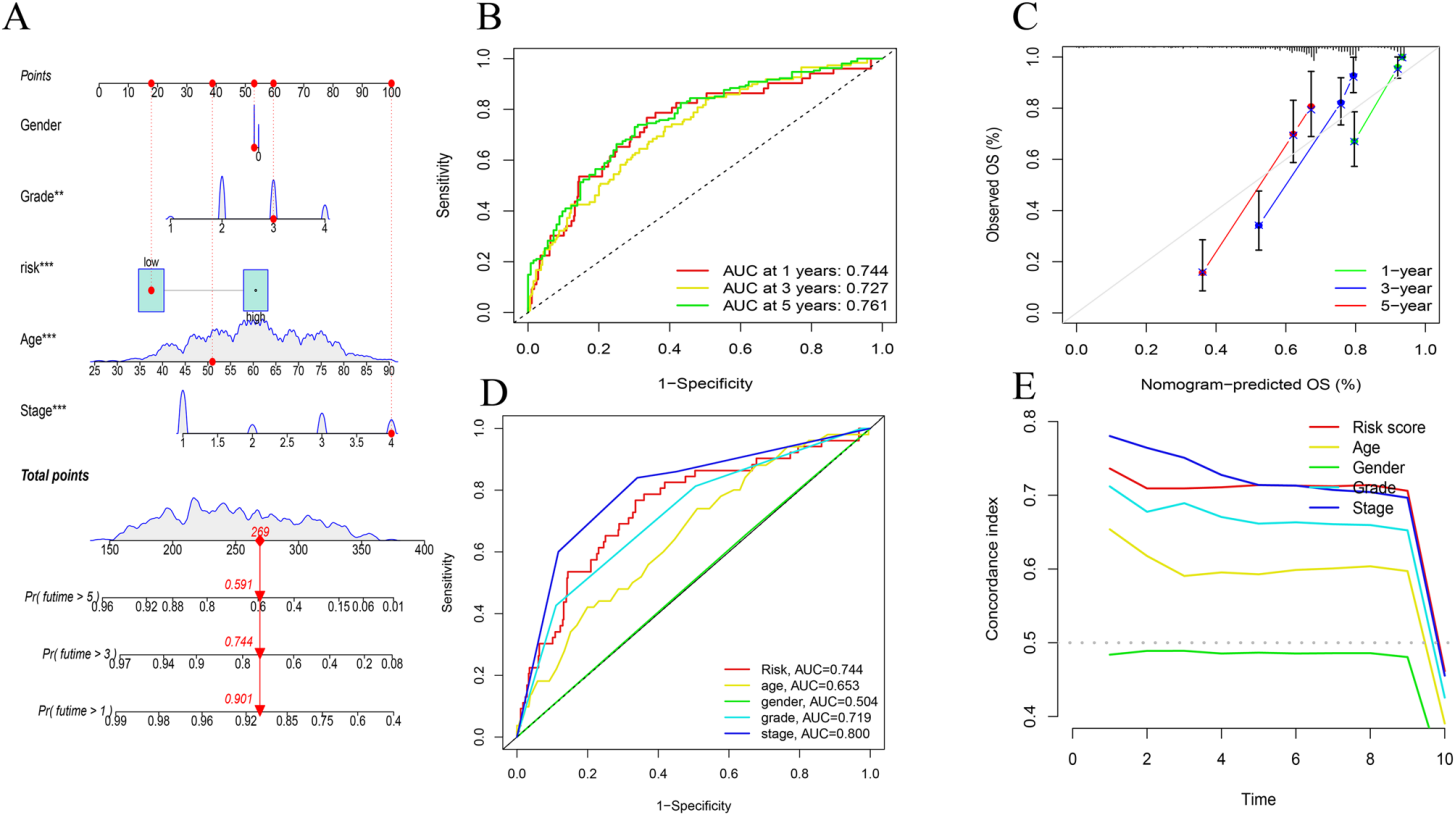
The predictive nomogram. (**A**) The predictive nomogram integrated with clinicopathological variables and risk classifications; (**B**) The AUC at 1-, 3-, 5-year of the m^7^G-associated lncRNAs signatures; (**C**) calibration curves of the nomogram; (**D**) the AUC of the m^7^G-associated lncRNAs signature and traditional clinical features; (**E**) the concordance index of the risk score and other clinical variables.

Gene Ontology enrichment analysis of differentially expressed m^7^G-related genes included various pathways of biological processes, cellular components, and molecular functions (Fig. 4). The risk calculation formula was y = -0.40*LINC01507 + 1.45*AL118508.1 + 0.33*AC093278.2 + 0.72*DUXAP8 + 1.15*AC084876.1 and the samples was divided patients into two risk group. A clear trend of prognostic distribution in two groups ccRCC patients (Fig. 5A). The scatter diagram shows low-risk patients have better survival status (Fig. 5B) and survival time (Fig. 5D) than another group. The heatmap shows the 5 m7G-related lncRNAs’s expression in ccRCC patients (Fig. 5C). The risk model was efficient at distinguishing patients according to the results of the risk level, survival status and time, expression of the five m^7^G-related lncRNAs for all patients (Fig. 6A,C,D,E) and a randomly selected subset of patients (Fig. 6B, F–H). The five m^7^G-related lncRNA prognostic signals and independent factors were used to create a mixed nomogram plot (Fig. 7A). The OS of the area under the curve (AUC) in the ROC plot predicted the survival of ccRCC patients at 1-, 3-and 5-year (Fig. 7B). Calibration curve was used to analyze the accuracy of the established model (Fig. 7C). The OS of the AUC predicted different factors (Fig. 7D). The consistency index (C-index) showed the established model outperformed traditional clinical factors in predicting ccRCC patients’s prognosis (Fig. 7E).

### Survival analysis and principal component analysis (PCA)

**Figure 8:**
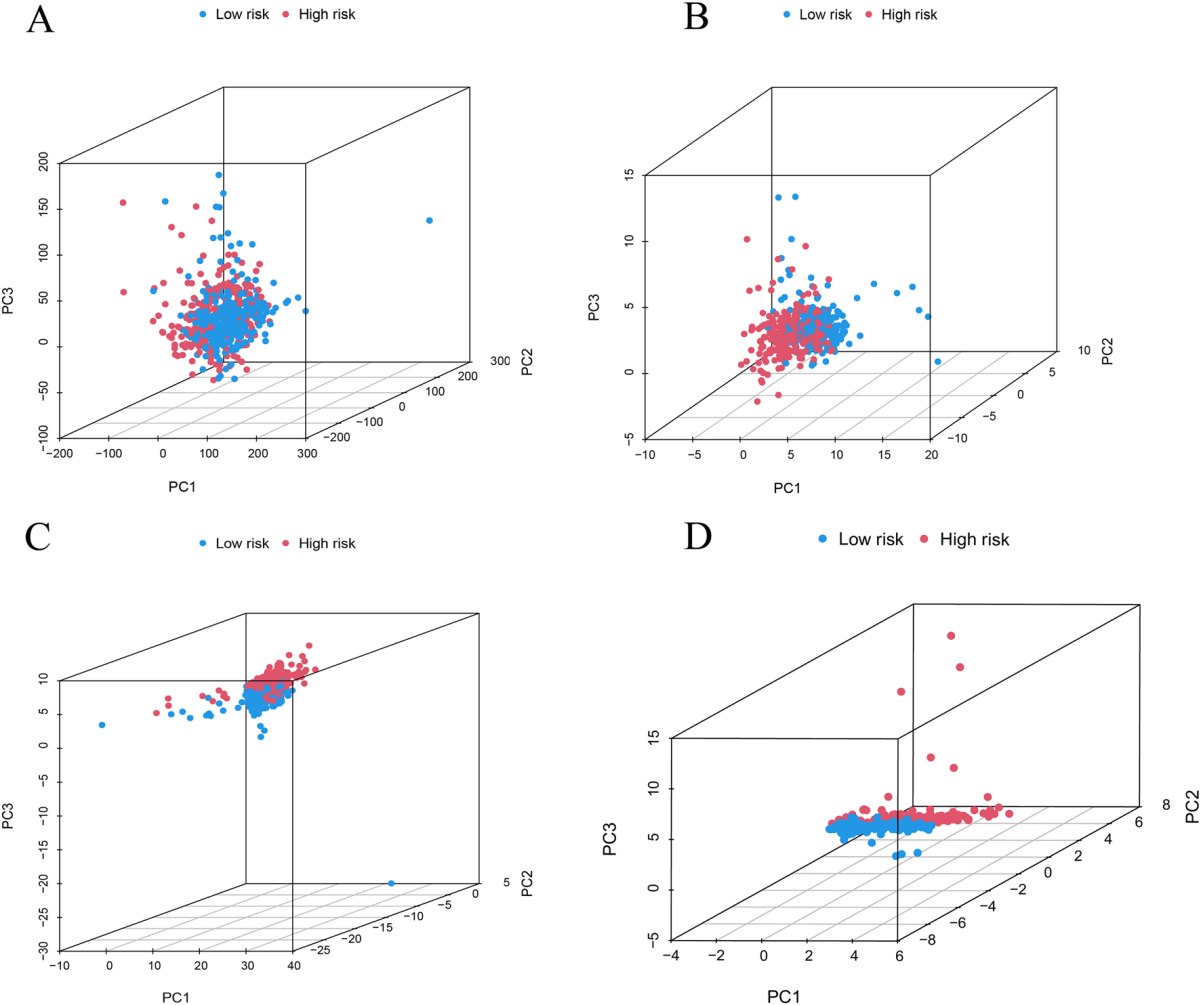
Principal component analysis. (**A**) The distribution of all genes; (**B**) the distribution of 29 m^7^G-related genes; (**C**) the distribution of 44 m^7^G-related lncRNAs; (**D**) the distribution of 5 m^7^G-related lncRNAs prognostic signature.

**Figure 9:**
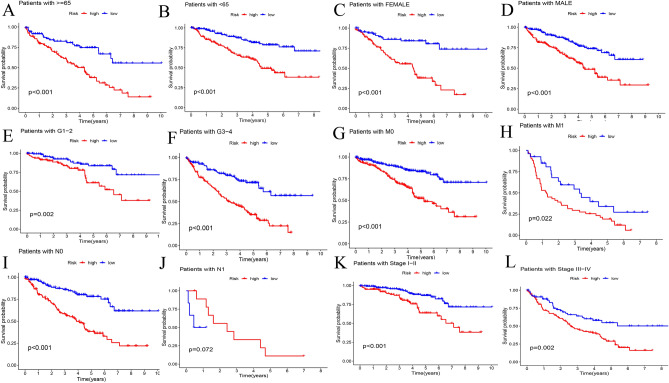
The Kaplan–Meier survival curves. (**A–L**) The Kaplan–Meier survival curves of overall survival rates in low- and high-risk groups based clinical factors.

The PCA diagram illustrate the whole gene expression of all ccRCC patients (Fig. 8A), 29 m7G genes (Fig. 8B), 44 m^7^G-associated lncRNAs (Fig. 8C), and five lncRNA risk models (Fig. 8D) in different risk groups. The five m^7^G-associated lncRNAs divided ccRCC patients into low- and high- risk levels more effectively than traditional clinical features. The K–M curves in the TCGA-KIRC dataset showed significantly better OS in ccRCC patients in the low-risk group according to age, sex, tumor-lymph node-metastasis stage, and grading (P < 0.001) (Fig. 9A–L).

### TIDE algorithm and IC_50_ for evaluating therapeutic responses

**Figure 10:**
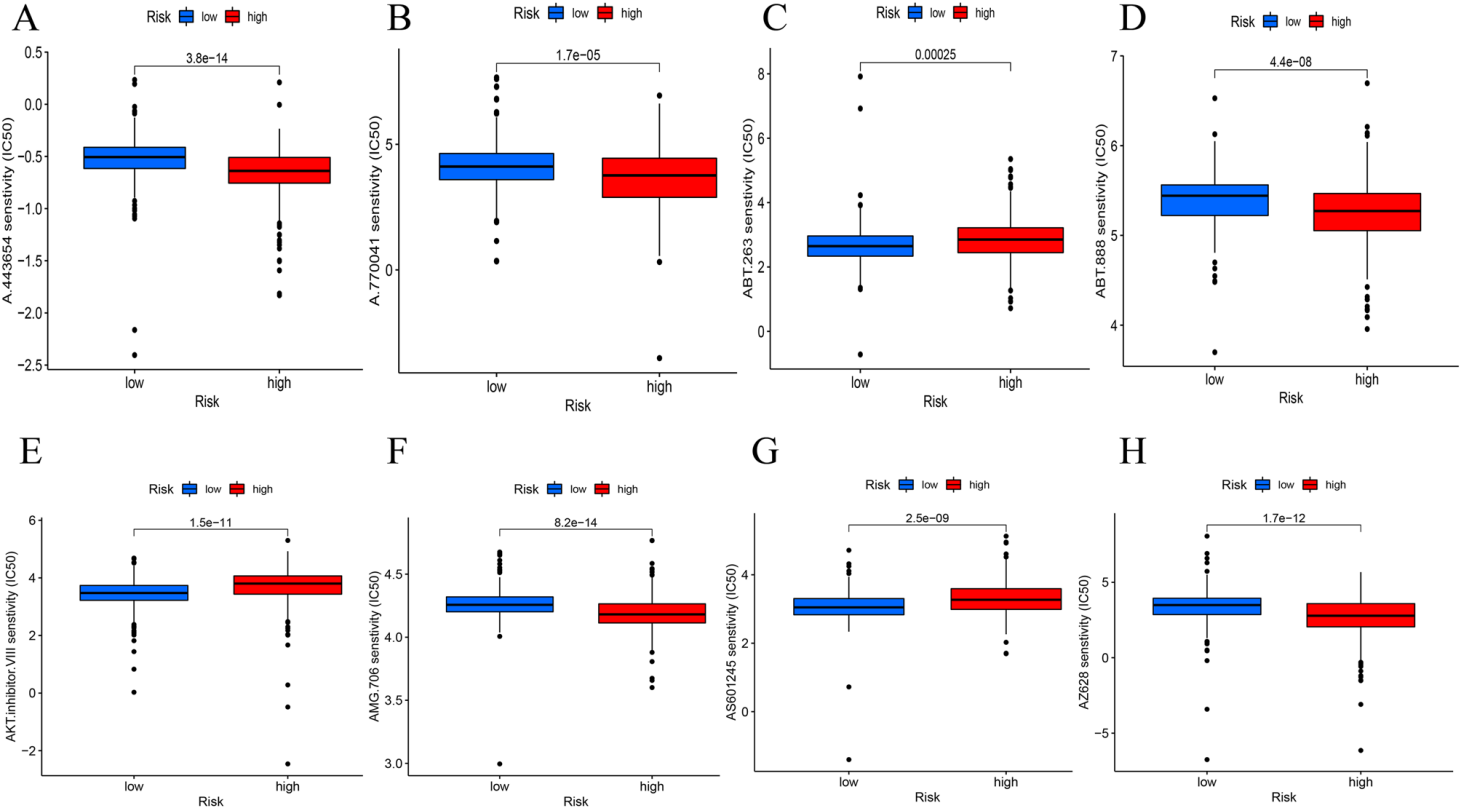
Comparison of drug sensitivity. (**A–H)** Estimated IC_50_ of potential drugs between low- and high-risk groups.

**Figure 11:**
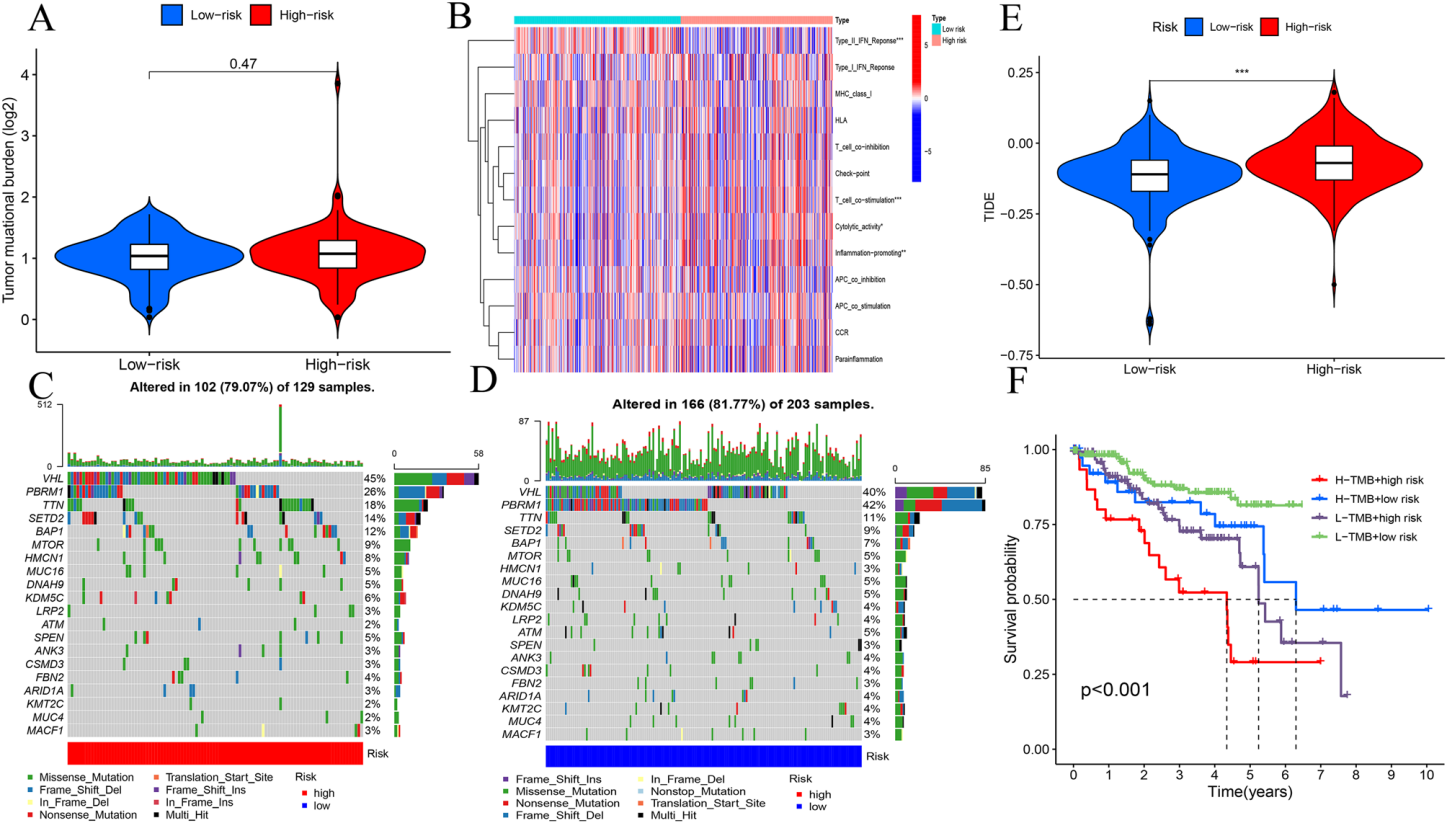
TMB correlated analysis and somatic mutation analysis. (**A**) TMB in low-risk and high-risk groups; (**B**) the heat map of immune response in two different risk groups; (**C,D**) the waterfall chart of the top 20 most frequently mutated genes in the different risk groups; (**E**) differences of immunotherapy risk score in the different risk groups; (**F**) Kaplan–Meier curves of OS in groups with various TMB and risk scores.

The risk model combined with a prediction algorithm to assess potential drug targets for ccRCC. Eight drugs could be used to further improve the treatment of ccRCC patients based on the estimated IC_50_ values (Fig. 10A–H). High-risk patients were more sensitive to A.443654, A.770041, ABT.888, AMG.706, and AZ628. Meanwhile, low-risk patients were more sensitive to ABT263, AKT inhibitor VIII, and AS601245. The tumor mutational burden (TMB) score failed to differ between the two risk groups (Fig. 11A). This indicated a poor association between the m^7^G-associated lncRNA classification index and TMB. The expression of indicators related to immune response and the mutation data were analyzed by MafTools software (Fig. 11B). The waterfall plot shows mutation information of 20 most frequently mutated genes in two groups (Fig. 11C, D). Low-risk ccRCC patients responded better to immunotherapy than high-risk patients based on prognostic models (Fig. 11E). This suggested that risk model may be helpful in predicting immunotherapy response. Lower TMB and risk result to higher survival rates (Fig. 11F). The results suggest that risk model may be more accurate than TMB in assessing the outcome of ccRCC patients.

### Verification of m^7^G-related lncRNA levels

**Figure 12:**
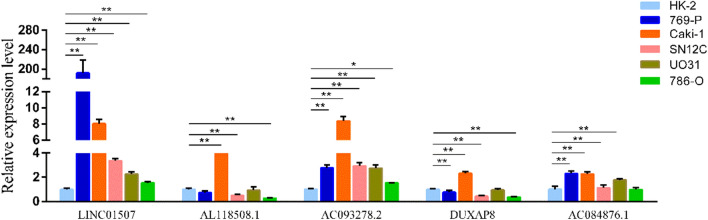
qRT-PCR results of five m^7^G-related lncRNAs in proximal tubular cell HK-2 and variable ccRCC cell lines. (*P < 0.05; **P < 0.01, Graphpad Prism 9.5.0).

LINC01507 and AC093278.2 levels were very high in all five ccRCC cell lines (p < 0.01) and were 2–180-fold higher than that in the proximal tubular HK-2 cell line according to qRT-PCR. AC084876.1 levels were elevated in all ccRCC cell lines (p < 0.01) except in 786-O cell lines (p > 0.05), although there was a less than threefold increase in other four ccRCC cell lines compared with HK-2 cells. The expression levels of AL118508.1 and DUXAP8 were upregulated in Caki-1 cell lines (p < 0.01). While the expression level of AL118508.1 was downregulated in SN12C and 786-O cells (p < 0.01), and there was no statistical difference between 769-P, UO31 cell lines and HK-2 cells (p > 0.05). DUXAP8 expression was downregulated in 769-P, SN12C, and 786-O cells (p < 0.01), but there was no statistical difference in the UO31 cell lines and HK-2 cells (p > 0.05) (Fig. 12). This illustrates the heterogeneity of ccRCC cells.

## Discussion

Kidney renal clear cell carcinoma is characterized by high recurrence and metastasis rates and relatively poor prognosis ^18,19^, which is associated with the complex heterogeneity of tumor cells ^20^. Recent studys contributed to identify stratification, treatment and prognosis of renal cancer by radiomics and artificial intelligence^2,3^. However, it is necessary to identify more efficient signatures of ccRCC to improve the early diagnosis and treatment. Notably, m^7^G RNA modification is involved in many physiological and pathological processes ^10^ of ccRCC and other tumors. LncRNAs initiate the disordered expression of protein-coding genes that act as pathogenic factors in ccRCC ^21^. However, current studies have mainly focused on the mechanisms of m^7^G and lncRNAs as independent factors in tumorigenesis. It is necessary to explore the relevance between m^7^G and lncRNAs to elucidate the potential pathogenic mechanisms of ccRCC.

This study identified 29 m7G genes from patients with ccRCC from the TCGA-KIRC database. Further analysis of patient clinical information was fetched to establish a prognostic model based on the five m^7^G-related lncRNAs. Gene Ontology enrichment analysis revealed that immune processes such as the humoral immune response, immunoglobulin complex, antigen binding, and complement activation were abundant and correlated with a previous report ^19^. Anaphylatoxin C5a ^22^ may promote tumor initiation and progression by modulating microenvironment during complement activation. Pentraxin-3(PTX3) ^23^ is involved in angiogenesis, proliferation and immune escape in cancer, and complement C1q plays an important role in the progression of ccRCC ^24^.

Depending on the risk model, each patient was assigned to high-risk or low-risk group; the training and validation groups were used to verify the reliability of the model. The reliability of the risk model was established through repeated verification. Notably, the prediction accuracy identified and visualized in ROC curves was better than that of traditional variables. PCA indicated that there were different distributions of patients based on various gene classifications. The OS rates showed obvious differences among the different risk groups based on several clinical characteristics.

Recently, lncRNAs have drawn increasing attention for their role in tumor initiation and progression. AC084876.1, AC093278.2, and DUXAP8 (screened m^7^G-related lncRNAs) showed pro-tumorigenic effects. A prognostic model based on 12 long noncoding RNAs has been developed by Ming et al. and confirmed the prognostic value of AL118508.1 ^19^. AC084876.1 plays a regulatory role in energy metabolism ^25^, and is a type of glycolysis-related lncRNA that is related to the occurrence and migration of RCC ^26^. Furthermore, acidification of the microenvironment during glycolysis promotes tumor cells infiltration and metastasis, not the growth of normal cells ^27,28^. AC093278.2 is an immune-related lncRNA that is considered to be a protective factor in ccRCC ^29^. Meanwhile, this study concluded that tumor heterogeneity leads to the driving effect of the same lncRNA in different cancer cell lines. Our qRT-PCR results indicated that AC093278.2 levels were very high in all five ccRCC cell lines compared with that in the HK-2 cell line. DUXAP8 is regarded as a novel metastasis-associated lncRNA that is linked to the invasive ability of RCC cells ^4^ and enhances RCC proliferation by downregulating miR-126 ^1^. Correspondingly, it regulates malignant phenotype and chemotherapy resistance of hepatocellular carcinoma through miR-584-5p/MAPK1/ERK Pathway Axis ^30^, promotes cell proliferation, migration, and invasion in multiple cancers through various pathways. Meanwhile, miR-223-3p mediates CXCR4 ^16^ in papillary thyroid carcinoma, silences EGR1 and RHOB^15^ in non-small-cell lung cancer, and epigenetically inhibits PLEKHO1 expression^31^ in gastric cancer. Unexpectedly, DUXAP8 showed higher expression levels only in Caki-1 cell lines which indicates the effect of tumor heterogeneity in cancer cells.

Immunotherapy plays a pivotal role in the treatment of ccRCC, therefore, it is of great value to analyze drug sensitivity based on IC_50_ assessment in two risk groups. Patients in the high-risk group were more sensitive to A.443654, A.770041, ABT.888, AMG.706, and AZ628, whereas those in the low-risk group were more sensitive to ABT263, AKT inhibitor VIII, and AS601245 according to the IC_50_. These results imply that ccRCC patients with different risks respond differently to these drugs, probably owing to differences in drug signaling pathways. These drugs include inhibitors of the AKT, LCK, PARP, VEGF, BRAF, Bcl-2/Bcl-xl, AKT, and JNK pathways. A.443654 is a specific inhibitor of AKT that inhibits viral replication in hepatitis B by downregulating Aurora A kinase ^32^. LCK is a druggable target gene associated with cancer cell invasion and metastasis. A.770041 inhibits LCK activity and blocks the invasion of oral cancer cells ^33^. ABT.888 acts as an inhibitor of PARP, reduces melanoma cell viability, and promotes apoptotic activity ^34^. AMG.706 is a potential agonist for MRGPRF and impedes tumor growth in vitro and in vivo ^35^. AZ628 is a hydrophobic Raf-kinase inhibitor, may enhance drug absorption during therapy of breast cancer ^36^. Combined treatment with TW-37 and ABT-263 (a Bcl-2 family protein inhibitor) inhibits the growth of RCC cells by synergistically inducing apoptosis through the mitochondrial pathway. Additionally, ERK signaling is activated after treatment with TW-37 and ABT.263 ^37^. AKT inhibitor further exacerbates cisplatin or TOPK inhibitor to induce apoptosis of renal tubular epithelial cells ^38^. AS601245 inhibits c-Jun N-terminal kinase (JNK), which regulates cancer cell apoptosis and survival ^39^.

This study utilized immune-related function analysis to differentiate the various expressed genes between the high- and low-risk groups. By inducing IFN-stimulated genes (ISGs), type I interferons (IFNs) play an important role in establishing and modulating the resistance of a host to microbial infection ^40^. Moreover, they facilitate cancer immunosurveillance, antitumor immunity, and antitumor efficacy of conventional cell death-inducing therapies ^41^. The binding of CD28 on T cells with CD80 and/or CD86 (CD80/86) ligands on antigen-presenting cells (APC) results in T cell co-stimulation. Immunization of mice with CD80/86-transduced tumors protects them from developing tumors to a certain extent ^42^. This study showed that there were higher immunotherapy risk scores in the high-risk group, suggesting that it has a more complicated immune microenvironment that could lead to a higher possibility of gene mutation compared with that of the low-risk group.

This study uncovered that gene mutations in ccRCC patients (including in VHL, PBRM1, and MTOR) were more frequent than other genes in waterfall plot. VHL is an important tumor suppressor that is lost in the majority of ccRCCs. Its regulatory pathway involves E3 ligase ^43^. PBRM1 mutations may cause ccRCC genomic instability and promote defects in the DNA repair pathway ^7^. Activation of mTOR promotes tumor growth and metastasis ^44^. Tumor mutational burden was not statistically different between the high- and low-risk groups. However, it was significantly associated with OS when combined with the different risk groups. High TIDE scores in the high-risk group imply a high probability of immune escape and poor outcomes during immunotherapy. Further studies should be conducted to clarify the potential mechanism by which ccRCC promotes therapeutic schedules.

In summary, a large amount of clinical data and survival prognosis information was obtained for ccRCC patients from the TCGA-KIRC database, and combined analysis of m^7^G and lncRNAs was performed to establish a prognostic prediction model. This model was internally validated and demonstrated good predictive properties for ccRCC prognosis. Our prognostic prediction model was better than traditional clinical features. Notably, we used KEGG database to analyze the biological roles of m^7^G-associated lncRNAs, which is beneficial to facilitate the efficient research. From a more professional point, bioinformatics has become a major discipline for handling massive datasets^45,46^. KEGG involved genomic information which contains metabolism, signal transduction of cellular processes^47,48^. Its practical values generate great influences on genomic information.

However, this study has some limitations. Firstly, this was a retrospective study wherein most (but not all) selection and recall biases were avoided due to imperfection of clinical data and heterogeneity of tumor microenvironment. In vivo and in vitro experiments are required to further validate our findings. Secondly, analyses of immune-related and drug sensitivity of the relevant lncRNAs did not find relevant external data in the available databases. The relationship between m^7^G and lncRNAs requires further investigation and validation. Furthermore, external validation of the prognostic risk model requires additional clinical sample sizes and relevant clinical trials. In addition, the m^7^G in this study is linear data, whereas chromatin in cells tends to be a 3D structure, and it remains unclear how these five m^7^G -associated lncRNAs affect gene regulation and the immune microenvironment in ccRCC. It is worthwhile to expect that machine learning is making rapid progress and that some more accurate algorithms and more optimized models will emerge, which may lead to more surprising results that make m^7^G -related lncRNAs shine in the diagnosis, treatment and prevention of ccRCC.

## Conclusion

A prognostic model based on the five m^7^G-associated lncRNAs was conducted. Bioinformatic analysis suggests that the five m^7^G-related lncRNA signatures act as risk factors for the development of RCC and exacerbate patient prognosis. Moreover, the tumor microenvironment of ccRCC may be regulated by m7G-related lncRNAs. This finding may be useful for further elucidating the potential mechanisms of how m^7^G-related lncRNAs may be related to ccRCC and may provide new ideas for individualized clinical treatment options.

## Data Availability

The datasets generated and analysed during the current study are publicly available in the TIDE database, and TCGA-KIRC database (https://portal.gdc.cancer.gov/).
